# Supplementary material for: Branched oncolytic peptides target HSPGs, inhibit metastasis, and trigger the release of molecular determinants of immunogenic cell death in pancreatic cancer
Source: Front Mol Biosci. 2024 Oct 2;11:1429163. doi: 10.3389/fmolb.2024.1429163 (PMC11479992; doi:10.3389/fmolb.2024.1429163)
Supplement: Supplementary file 1 [file DataSheet1.PDF]

SUPPLEMENTARY MATERIAL

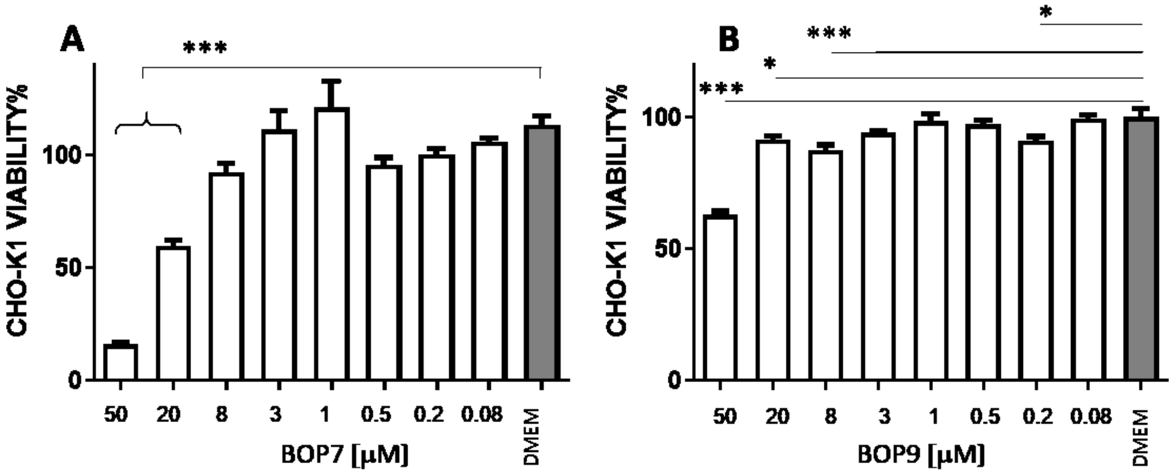

Supplementary Material 1. Viability of CHO-K1 cell line after treatment with BOP7 (A) IC50 5.0 x 10^{-3} M (n=5) IC50 and BOP9(B) IC50: 1.6 x 10^{-2} M (n=5). Data were analysed with one way ANOVA, Dunnet post test (*** $p < 0.0001$, * $p< 0.05$). IC50 were calculated in a non-linear fit variable slope. All graphs are obtained with GraphPad Prism 5.
